# Synthesis, X-ray Structure, Optical, and Electrochemical Properties of a White-Light-Emitting Molecule

**DOI:** 10.3390/ma9010048

**Published:** 2016-01-14

**Authors:** Jiun-Wei Hu, Ying-Hsuan Wu, Hsing-Yang Tsai, Kew-Yu Chen

**Affiliations:** Department of Chemical Engineering, Feng Chia University, Taichung 40724, Taiwan; p0400205@fcu.edu.tw (J.-W.H.); m0306953@fcu.edu.tw (Y.-H.W.); p0156676@fcu.edu.tw (H.-Y.T.)

**Keywords:** ESIPT, tautomer, white-light-emitting molecules, Stokes shift, X-ray diffraction, DFT calculations

## Abstract

A new white-light-emitting molecule (**1**) was synthesized and characterized by NMR spectroscopy, high resolution mass spectrometry, and single-crystal X-ray diffraction. Compound **1** crystallizes in the orthorhombic space group *Pnma*, with *a* = 12.6814(6), *b* = 7.0824(4), *c* = 17.4628(9) Å, α = 90°, β = 90°, γ = 90°. In the crystal, molecules are linked by weak intermolecular C-H···O hydrogen bonds, forming an infinite chain along [100], generating a *C*(10) motif. Compound **1** possesses an intramolecular six-membered-ring hydrogen bond, from which excited-state intramolecular proton transfer (ESIPT) takes place from the phenolic proton to the carbonyl oxygen, resulting in a tautomer that is in equilibrium with the normal species, exhibiting a dual emission that covers almost all of the visible spectrum and consequently generates white light. It exhibits one irreversible one-electron oxidation and two irreversible one-electron reductions in dichloromethane at modest potentials. Furthermore, the geometric structures, frontier molecular orbitals (MOs), and the potential energy curves (PECs) for **1** in the ground and the first singlet excited state were fully rationalized by density functional theory (DFT) and time-dependent DFT calculations. The results demonstrate that the forward and backward ESIPT may happen on a similar timescale, enabling the excited-state equilibrium to be established.

## 1. Introduction

Excited-state intramolecular proton transfer (ESIPT) molecules have been drawing significant attention due to their unusual optical properties [1,2,3,4,5,6]. An ESIPT reaction usually involves the transfer of a hydroxyl proton to an acceptor such as carbonyl oxygen (imine nitrogen) through a pre-existing hydrogen bonding system [7,8,9]. Molecules that exhibit ESIPT in the ground state exist predominantly as enol (E) forms; however, upon photoexcitation, they undergo tautomerization into keto forms (E* → K*) via an ultra-fast and irreversible ESIPT process occurring in the sub-picosecond time domain [10]. The resulting proton-transfer tautomer is totally different in structure and electronic configuration from its corresponding ground state, that is, a large Stokes shifted K* → K fluorescence. This unique optical property has many potential applications, typical examples of which are probes for solvation dynamics and biological environments [11,12,13,14], fluorescence microscopy imaging [15], photochromic materials [16], chemosensors [17,18,19,20,21], nonlinear optical materials [22], near-infrared fluorescent dyes [23], and organic light-emitting diodes (OLEDs) [24,25,26].

**Figure 1:**
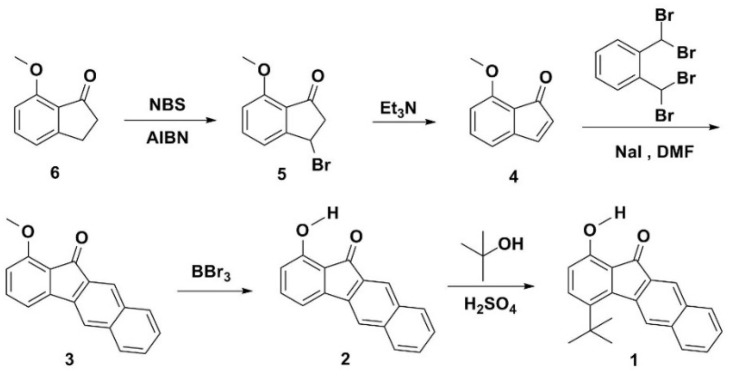
The synthetic route of 4-*tert*-Butyl-1-hydroxy-11*H*-benzo[b]fluoren-11-one (**1**). AIBN: 2,2′-azobisisobutyronitrile; NBS: *N*-bromosuccinimide.

White organic light-emitting diodes (WOLEDs) are of great interest due to their wide application as backlights for large-area flat-panel displays and solid-state lighting [27,28,29,30,31,32]. An ideal white emission should be composed of the three primary colors and cover the whole visible spectral range. Most of the WOLEDs reported so far are required to fabricate multi-emission-layer devices, in which each layer emits a primary color, thus achieving white emission [33,34,35,36,37]. Compared to WOLEDs with multiple-emitting components, a single-molecule-based WOLED could provide easy fabrication with excellent stability and color reproducibility [38,39]. Recently, with an aim to create white-light-emitting single molecules for WOLEDs, two research groups have made different approaches based on novel ESIPT chromophores. On the one hand [40], Park and colleagues have fabricated WOLEDs based on a single molecular dyad consisting of two ESIPT molecules. On the other hand [41], Tang and co-workers have demonstrated a single-molecule-based WOLED based on a new ESIPT-based white-light-emitting small molecule **2** (Figure 1). Taking advantage of an adiabatic ESIPT reaction, they show that broad blue emission from the excited starting material and broad orange emission from an ESIPT molecule **2** (Figure 1) combined to generate white light as if there are two molecules present in the sample. However, due to its relatively low overall fluorescence quantum yield, the external quantum efficiency (EQE) of the device is 0.11%, which is only decent compared with the work (EQE: 0.10%) mentioned earlier utilizing multi-ESIPT moieties [42]. In an effort to expand the scope of the **2**-based molecules available for designing systems for high performance single-molecule-based WOLEDs, we herein report the synthesis of a new derivative of **2** with a bulky *tert*-butyl moiety attached to the phenol ring; that is, 4-*tert*-butyl-1-hydroxy-11*H*-benzo[b]fluoren-11-one (**1**). Its X-ray structure, as well as optical and electrochemical properties and time-dependent density functional theory (TD-DFT) calculations, are also reported. The results offer the potential to synthesize white-light-emitting small molecules with extended molecular architectures and optical properties.

## 2. Experimental Section

### 2.1. General

The starting materials such as 7-methoxy-2,3-dihydro-1*H*-inden-1-one (**6**), 2,2′-azobisisobutyronitrile (AIBN), *N*-bromosuccinimide (NBS), α,α,α′α′-terarbromo-*o*-xylene, triethylamine, sodium iodide, boron tribromide, and *tert*-butyl alcohol were purchased from Merck (Whitehouse Station, NJ, USA), ACROS (Pittsburgh, PA, USA), and Sigma-Aldrich (St. Louis, MO, USA). Solvents were distilled freshly according to standard procedure. Column chromatography was performed using silica gel Merck Kieselgel *si* 60 (40–63 mesh). ^1^H and ^13^C-NMR spectra were recorded in CDCl_3_ on a Bruker 400 MHz NMR spectrometer (Bruker, Palo Alto, CA, USA). Mass spectra were recorded on a VG70-250S mass spectrometer (Hitachi, Tokyo, Japan). The absorption and emission spectra were measured using a Jasco V-570 UV-Vis spectrophotometer (Jasco, Tokyo, Japan) and Hitachi F-7000 fluorescence spectrophotometer (Hitachi, Tokyo, Japan), respectively. The single-crystal X-ray diffraction data were collected on a Bruker Smart 1000CCD area-detector diffractometer (Bruker, Billerica, MA, USA). Cyclic voltammetry (CV) was performed with a CH instruments (CH instruments, Austin, TX, USA) at a potential rate of 200 mV/s in a 0.1 M solution of tetrabutylammonium hexafluorophosphate (TBAPF_6_) in dichloromethane. Platinum and Ag/AgNO_3_ electrodes were used as counter and reference electrodes, respectively.

### 2.2. Synthesis

#### 2.2.1. Synthesis of 3-Bromo-7-methoxy-2,3-dihydro-1*H*-inden-1-one (**5**)

7-Methoxy-2,3-dihydro-1*H*-inden-1-one (1.0 g, 6.2 mmol) and *N*-bromosuccinimide (1.2 g, 6.8 mmol) were dissolved in 20 mL of CCl_4_ and 12 mg (1 mol %) of 2,2′-azobisisobutyronitrile (AIBN) was added. The mixture was slowly stirred and heated to 80 °C for 2 h. After cooling, the mixture was poured into cold water, extracted with CH_2_Cl_2_, and dried with anhydrous MgSO_4_. After the solvent was removed, the crude product was purified by silica gel column chromatography with eluent ethyl acetate/*n*-hexane (1/4) to afford **5** (1.1 g, 74%). ^1^H-NMR (CDCl_3_, ppm) 7.62 (t, *J* = 8.5 Hz, 1H), 7.20 (d, *J* = 8.0 Hz, 1H), 6.87 (d, *J* = 8.5 Hz, 1H), 5.52 (dd, *J*_1_ = 7.0 Hz, *J*_2_ = 2.0 Hz, 1H), 3.92 (s, 3H), 3.32 (dd, *J*_1_ = 18.5 Hz, *J*_2_ = 7.0 Hz, 1H), 2.97 (dd, *J*_1_ = 18.5 Hz, *J*_2_ = 2.0 Hz, 1H); MS (FAB) *m*/*z* 240 (M + H)^+^. HRMS (High Resolution Mass Spectrum) calculated for C_10_H_10_BrO_2_ 240.9864, found 240.9866.

#### 2.2.2. Synthesis of 7-Methoxy-1*H*-inden-1-one (**4**)

3-Bromo-7-methoxy-2,3-dihydro-1*H*-inden-1-one (1.0 g, 4.1 mmol) in CCl_4_ was cooled in an ice bath, and 1.93 mL (1.4 g, 14.0 mmol) of triethylamine was added slowly. The reaction mixture was allowed to warm to room temperature and stir overnight. After the solvent was removed, the crude product was purified by silica gel column chromatography with eluent ethyl acetate/*n*-hexane (1/4) to afford **4** (0.63 g, 95%). ^1^H-NMR (CDCl_3_, ppm) 7.42 (d, *J* *=* 6.0 Hz, 1H), 7.34 (t, *J* *=* 8.0 Hz, 1H), 6.85 (d, *J* *=* 8.8 Hz, 1H), 6.70 (d, *J* *=* 8.0 Hz, 1H), 5.84 (d, *J* *=* 6.0 Hz, 1H), 3.95 (s, 3H); ^13^C-NMR (100 MHz, CDCl_3_, ppm) 196.57, 156.50, 147.38, 147.03, 144.48, 136.02, 127.70, 115.65, 114.67, 55.89; MS (FAB) *m/z* 161 (M + H)^+^. HRMS calculated for C_10_H_9_O_2_ 161.0603, found 161.0608.

#### 2.2.3. Synthesis of 7-Methoxy-1*H*-inden-1-one (**3**)

A mixture of solution of α,α,α’α’-terarbromo-*o*-xylene (1.3 g, 3.1 mmol), 7-methoxy-1*H*-inden-1-one (0.5 g, 3.1 mmol), sodium iodide (1.8 g, 12 mmol) and dry *N,N*-dimethylformamide (30 mL) was stirred at 65 °C for 24 h. The reaction mixture was poured into cold water (70 mL) containing sodium bisulfite (1.0 g). The yellow precipitate was purified by silica gel column chromatography with eluent ethyl acetate/*n*-hexane (1/4) to afford **3** (0.73 g, 90%). ^1^H-NMR (CDCl_3_, ppm) 8.14 (s, 1H), 7.79~7.88 (m, 3H), 7.43~7.54 (m, 3H), 7.31 (d, *J* *=* 7.6 Hz, 1H), 6.85 (d, *J* *=* 8.4 Hz, 1H), 3.99 (s, 3H); ^13^C-NMR (100 MHz, CDCl_3_, ppm) 191.06, 158.54, 146.83, 137.56, 136.96, 136.50, 133.76, 133.10, 130.58, 128.68, 128.59, 126.78, 125.01, 122.74, 119.03, 113.26, 112.37, 55.89; MS (FAB) *m*/*z* 261 (M + H)^+^. HRMS calculated for C_18_H_13_O_2_ 261.0916, found 261.0918.

#### 2.2.4. Synthesis of 1-Hydroxy-11*H*-benzo[b]fluoren-11-one (**2**)

1-Methoxy-11*H*-benzo[b]fluoren-11-one (300 mg, 1.1 mmol) was dissolved in 10 mL of dichloromethane in a 50 mL round-bottom flask, and the flask was placed in an ice bath at 0 °C. A solution of boron tribromide (0.25 mL, 1.0 M solution in dichloromethane) was added carefully to the stirred solution under a nitrogen atmosphere. After 4 h, the reaction was cooled and the reaction mixture was then hydrolyzed by carefully shaking it with 10 mL of water and extracted twice with 10 mL of dichloromethane. The combined organic phases were then dried over magnesium sulfate, filtered, and evaporated *in vacuo*; the crude product was purified by silica gel column chromatography with eluent ethyl acetate/*n*-hexane (1/10) to afford **2** (269 mg, 95%). ^1^H-NMR (CDCl_3_, ppm) 8.64 (s, 1H), 8.07 (s, 1H), 7.84 (d, *J* *=* 7.5 Hz, 1H), 7.78 (s, 1H), 7.77 (d, *J* *=* 9.0 Hz, 1H), 7.54 (t, *J* *=* 7.5 Hz, 1H), 7.38~7.46 (m, 2H), 7.15 (d, *J* *=* 7.0 Hz, 1H), 6.76 (d, *J* *=* 8.0 Hz, 1H); ^13^C-NMR (100 MHz, CDCl_3_, ppm) 195.51, 157.66, 144.25, 137.99, 137.81, 136.74, 133.41, 132.68, 130.78, 129.13, 128.85, 127.08, 125.54, 120.16, 120.01, 117.40, 113.00; MS (FAB) *m*/*z* 247 (M + H)^+^. HRMS calculated for C_17_H_11_O_2_ 247.0759, found 247.0755.

#### 2.2.5. Synthesis of 4-*tert*-Butyl-1-hydroxy-11*H*-benzo[b]fluoren-11-one (**1**)

1-Hydroxy-11*H*-benzo[b]fluoren-11-one (200 mg, 0.8 mmol) was added to *tert*-butyl alcohol (5 mL, 52.7 mmol). Sulfuric acid (0.5 mL, 9.3 mmol) was then added dropwise over 1 min and the mixture was allowed to stir for 2 h. H_2_O (10 mL) was added to the flask and the mixture allowed to stir for 10 min. The mixture was extracted with dichloromethane and dried with magnesium sulfate; the crude product was purified by silica gel column chromatography with eluent ethyl acetate/*n*-hexane (1/6) to afford **1** (197 mg, 80%). Yellow parallelepiped-shaped crystals suitable for the crystallographic studies reported here were isolated over a period of five weeks by slow evaporation from a dichloromethane solution. ^1^H-NMR (CDCl_3_, ppm) 9.66 (s, 1H), 8.22 (s, 1H), 8.18 (s, 1H), 7.89 (m, 2H), 7.58 (m, 3H), 6.78 (d, *J* *=* 8.8 Hz, 1H), 1.60 (s, 9H); ^13^C-NMR (100 MHz, CDCl_3_, ppm) 196.4, 156.9, 141.1, 140.0, 138.0, 136.5, 136.1, 133.2, 132.3, 130.1, 129.5, 129.0, 128.1, 127.5, 125.0, 121.2, 117.4, 34.6, 30.4; MS (FAB) *m*/*z* 303 (M + H)^+^. HRMS calculated for C_21_H_1__9_O_2_ 303.1385, found 303.1387.

### 2.3. Crystal Structural Determination

A single crystal of **1** with dimensions of 0.56 mm × 0.40 mm × 0.25 mm was selected. The lattice constants and diffraction intensities were measured with a Bruker Smart 1000CCD area detector radiation (*λ* = 0.71073 Å) at 297(2) K (Bruker, Billerica, MA, USA). An *ω*-2*θ* scan mode was used for data collection in the range of 2.83° ≤ *θ* ≤ 29.16°. A total of 6225 reflections were collected and 2013 were independent (*R*_int_ = 0.0961), of which 1340 were considered to be observed with *I* > 2*σ*(*I*) and used in the succeeding refinement. The structure was solved by direct methods with SHELXS-97 [43] and refined on *F*^2^ by full-matrix least-squares procedure with Bruker SHELXL-97 packing (Bruker, Billerica, MA, USA) [44]. All non-hydrogen atoms were refined with anisotropic thermal parameters. The hydrogen atoms refined with riding model position parameters isotropically were located from difference Fourier map and added theoretically. At the final cycle of refinement, *R* = 0.0752 and *wR* = 0.2021 (*w* = 1/[*σ*^2^(*F_o_*^2^) + (0.1308*P*)^2^ + 0.0406*P*], where *P* = (*F_o_*^2^ + 2*F_c_*^2^)/3). *S* = 1.055, (Δ/*σ*)_max_ = 0.014, (Δ/*ρ*)_max_ = 0.345 and (Δ/*ρ*)_min_ = −0.259 e/Å^3^.

### 2.4. Steady State Spectral Measurements

All the spectral measurements were done at 10^−5^ M concentration of solute in order to avoid aggregation and self-quenching. The fluorescence quantum yield of **1** and **2** in ethyl acetate was measured relative to quinine sulphate in 1 M sulphuric acid (Φ_f_ *=* 0.57) as secondary standard [45] and calculated on the basis of the following equation:

(1) $$\Phi_{f}=\Phi_{f}^{0}\frac{n^{2}A_{0}\int^{\text{​}}I_{f}\left(\lambda_{f}\right)d\lambda_{f}}{n_{0}^{2}A\int^{\text{​}}I_{f}^{0}\left(\lambda_{f}\right)d\lambda_{f}}$$

where n_0_ and n are the refractive index of the solvents; A_0_ and A are the absorbances; $\Phi_{f}$ and $\Phi_{f}^{0}$ are the fluorescence quantum yields; and the integrals denote the area of the fluorescence band for the standard and the sample, respectively.

### 2.5. Computational Methods

The Gaussian 03 program (Gaussian, Pittsburgh, PA, USA) was used to perform the *ab initio* calculation on the molecular structure [46]. Full geometry optimizations of compound **1** were carried out with the 6-31G** basis set to the B3LYP functional. The hybrid DFT functional B3LYP has proven to be a suitable DFT functional to describe hydrogen bond [47]. Vibrational frequencies were also performed to check whether the optimized geometrical structures for **1** were at energy minima, transition states, or higher order saddle points. After obtaining the converged geometries, the TD-B3LYP/6-31G** was used to calculate the vertical excitation energies. Emission energies were obtained from TDDFT/B3LYP/6-31G** calculations performed on S_1_ optimized geometries. The phenomenon of photo-induced proton transfer (PT) reaction in **1** can be most critically addressed and assessed by evaluating the potential energy curve (PEC) for the PT reaction. For the S_0_ state, all of the other degrees of freedom are relaxed without imposing any symmetry constraints. The excited-state (S_1_) PEC for the ESIPT reaction in **1** has been constructed on the basis of TD-DFT optimization method.

## 3. Results and Discussion

### 3.1. Synthesis

Figure 1 depicts the chemical structures and the synthetic routes of white-light-emitting small molecules **1** and **2**. The synthesis of **1** started from a bromination of 7-methoxy-1-indanone (**6**), followed by the elimination of **5**, giving a dienophile **4**. The naphthalene ring can then be fused onto the C(2)-C(3) double bond by placing **4** through a reaction with α,α,α’α’-tetrabromo-*o*-xylene [41], yielding **3**. Subsequently, deprotection of **3** with BBr_3_ produced compound **2**. Finally, the regioselective alkylation at the 4-position of **2** was executed by the reaction of **2** with *tert*-butyl alcohol and sulfuric acid, giving **1** with an overall product yield of 65%. The presence of a single *tert*-butyl group of **1** can be verified by the presence of a signal at δ 1.42 ppm (9H, singlet) and eight signals at δ 7.0–8.2 ppm (8H) in the ^1^H-NMR spectrum. To confirm its structure, a single crystal of **1** was obtained from a dichloromethane solution, and the molecular structure was determined by X-ray diffraction analysis. Additionally, its X-ray structure is compared with that of **2**.

### 3.2. Hydrogen Bond Studies

**Figure 2:**
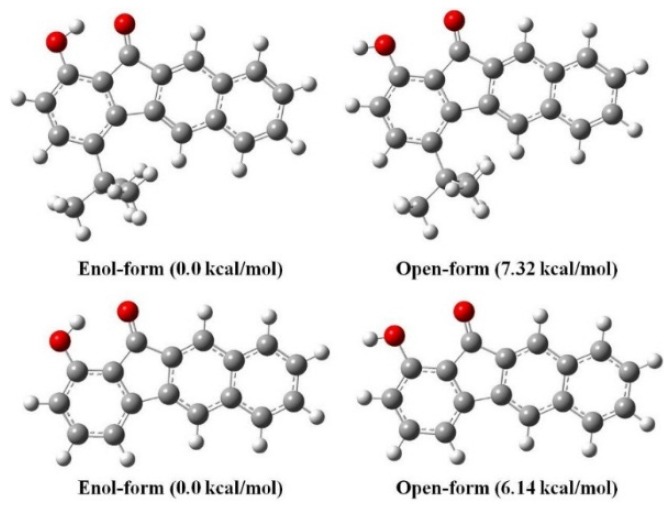
Computed energies of different conformers of **1** (top) and **2** (bottom) are specified relative to the respective enol-form (DFT/B3LYP/6-31G**).

The dominance of an enol-form for **1** and **2**, namely the intramolecular hydrogen-bond formation between O(2)-H(2A) and O(1), is firmly evidenced by a combination of ^1^H-NMR and X-ray single-crystal analyses. In the ^1^H-NMR studies, the existence of an intramolecular hydrogen bond between O(2)-H(2A) and O(1) is supported by the observation of a significant downfield shift of the proton peak at δ > 8 ppm (in dry CDCl_3_) for both compounds **1** (9.66 ppm) and **2** (8.64 ppm). The hydrogen bonding energy (Δ*E* in kcal/mol) of **1** and **2** can be calculated by introducing Schaefer’s correlation [48], expressed as Δδ = (−0.4 ± 0.2) + Δ*E*, where Δδ is given in parts per million for the difference between chemical shift in the O-H peak of **1** and **2** and that in phenol (δ 4.29). Accordingly, the hydrogen-bonding energy is calculated to be **1** (5.77 ± 0.2 kcal/mol) > **2** (4.75 ± 0.2 kcal/mol) and is in good agreement with the theoretical calculations (Figure 2). Note that the substitution of the hydrogen atom at the 4-position in **2** by a bulky *tert*-butyl substituent, forming **1**, seems to increase the acidity of phenol (O(2)-H(2A)) through an inductive effect. As a result, compound **1** shows a small downfield shift of the O(2)-H(2A) proton, and hence, a stronger hydrogen bond relative to **2**.

### 3.3. X-ray Structures

**Figure 3:**
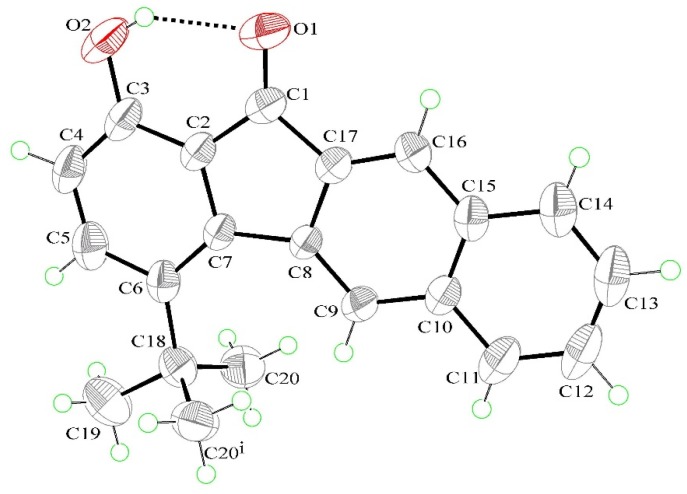
Displacement ellipsoid representation of **1** with the labelling scheme. The ellipsoids are drawn at the 50% probability level and the H atoms are drawn as spheres of arbitrary radii. The black dashed line denotes the intramolecular O-H···O hydrogen bond.

Compound **1** crystallizes in the orthorhombic space group *Pnma*, whereas the closely related compound **2** crystallizes in the monoclinic space group *P*2_1_/c (Table 1). Figure 3 shows the ORTEP (Oak Ridge Thermal Ellipsoid Plot) diagram of **1**. The molecule is completely planar (except for *tert*-butyl substituent), as indicated by the key torsion angles (Table 2). Compound **1**, as well as compound **2**, possesses an intramolecular O-H···O hydrogen bond, which generates an S(6) ring motif. The dihedral angle between the mean plane of the S(6) ring and the mean plane of the phenol ring is 0.0°. This, together with 2.786(3) Å of O(2)···O(1) distance and 145° of O(2)-H(2A)···O(1), strongly supports the S(6) ring formation. The O(2)···O(1) distance of **1** is slightly shorter than that of **2** (2.879(3) Å of O(2)···O(1) distance and 143° of O(2)-H(2A)-O(1)), consistent with the hydrogen-bonding strength estimated from ^1^H-NMR measurements (*vide supra*). Moreover, the longer O(2)···O(1) distance in **1** (**2**) than that (O(2)-O(1) < 2.7 Å) in most other ESIPT molecules supports that compound **1** (**2**) has a weaker intramolecular hydrogen bond. This is possible due to the fact that the carbonyl oxygen O(1) sits at the five-membered-ring cyclopenta-2,4-dienone moiety (Figure 3), such that the ∠O(2)-H(2A)-O(1) angle is expected to be deviated from 120°, a perfect six-membered-ring hydrogen-bonding formation. This viewpoint is confirmed by the ∠O(2)-H(2A)-O(1) angle of 145° (143°), according to the X-ray structure analysis. Note that compound **1** (**2**) has a weaker intramolecular hydrogen bond than most other ESIPT chromophores [49,50], which may account for its unique dual emission feature (*vide infra*).

**Table 1:** Crystallographic data for compounds **1** and **2**.

Compound	1	2
Chemical Formula	C_21_H_17_O_2_	C_17_H_10_O_2_
Formula Weight	301.35	246.25
Crystal System	Orthorhombic	Monoclinic
Space Group	*Pnma*	*P*2_1_/c
*a* (Å)	12.6814(6)	12.474(2)
*b* (Å)	7.0824(4)	6.4401(12)
*c* (Å)	17.4628(9)	15.601(3)
α (°)	90	90
β (°)	90	109.188(3)
γ (°)	90	90
Volume (Å^3^)	1568.42(14)	1183.6(4)
Z	4	4
D_calc_ (g cm^−3^)	1.276	1.382
*μ* (mm^−1^)	0.081	0.090
F_000_	636	512
Crystal Size (mm^3^)	0.56 × 0.40 × 0.25	0.42 × 0.22 × 0.12
*θ* range (°)	2.83–29.16	1.73–26.00
Index ranges	−9 ≤ h ≤ 17	−14 ≤ h ≤ 15
	−9 ≤ k ≤ 9	−7 ≤ k ≤ 7
	−13 ≤ l ≤ 23	−19 ≤ l ≤ 16
Reflections Collected	6225	6331
Independent Reflections (*R*_int_)	2013 (0.0961)	2310 (0.0445)
Refinement Method on F^2^	Full-matrix least-squares	Full-matrix least-squares
GOF on F^2^	1.055	1.014
*R*_1_ [*I* > 2*σ* (*I*)]	0.0752	0.0571
*wR*_2_ [*I* > 2*σ* (*I*)]	0.2021	0.1488
*R*_1_ (All Data)	0.1017	0.1047
*wR*_2_ (All Data)	0.2334	0.1877
Residual (*e* Å^−3^)	0.345 and −0.259	0.235 and −0.183

**Table 2:** Comparison of the experimental and optimized geometric parameters of **1** (Å and °). DFT: Density Functional Theory.

Compound 1	X-ray	DFT
**Bond Lengths (Å)**		
O(2)-C(3)	1.352(4)	1.345
O(1)-C(1)	1.229(3)	1.237
C(1)-C(2)	1.459(4)	1.458
C(2)-C(3)	1.391(4)	1.395
C(7)-C(8)	1.505(3)	1.509
C(8)-C(9)	1.364(3)	1.376
C(11)-C(12)	1.367(5)	1.380
C(18)-C(19)	1.548(4)	1.546
**Bond Angles (°)**		
O(1)-C(1)-C(2)	126.0(3)	125.9
O(2)-C(3)-C(4)	121.1(3)	121.3
C(1)-C(2)-C(3)	124.8(3)	123.5
C(2)-C(7)-C(8)	106.1(2)	105.6
C(9)-C(10)-C(11)	121.1(3)	121.1
C(13)-C(14)-C(15)	120.3(3)	120.7
**Torsion Angles (°)**		
O(1)-C(1)-C(2)-C(3)	0.0	0.0
O(2)-C(3)-C(4)-C(5)	0.0	0.0
C(8)-C(9)-C(10)-C(11)	0.0	0.0
C(14)-C(15)-C(16)-C(17)	0.0	0.0

**Figure 4:**
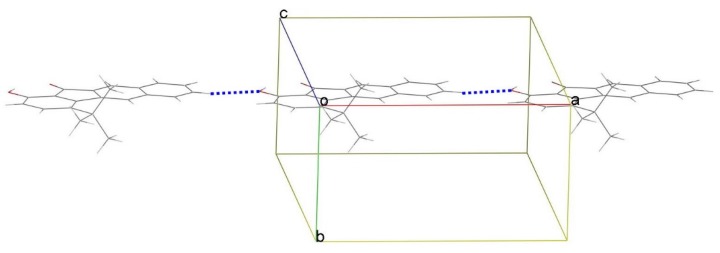
A packing view of **1**, viewed along the *c* axis. Blue dashed lines denote intermolecular C-H···O hydrogen bonds.

Figure 4 shows the molecular packing of **1** in the crystal unit cell. The crystal structure is further stabilized by weak intermolecular C-H···O hydrogen bonds (2.53 Å of H(12A)···O(2) distance and 151° of C(12)-H(12A)-O(2), symmetry code: 1 + X, Y, Z), forming an infinite chain along [100] and generating a *C*(10) motif. Careful examination of the crystal structure also depicts that there is no substantial π-π stacking between the tetracyclic plane and its adjacent one. As a result, we can ascertain that the bulky *tert*-butyl substituent not only increases the solubility of **1** compared with **2**, but also reduces intermolecular contact and aggregation.

### 3.4. Photophysical Properties

**Figure 5:**
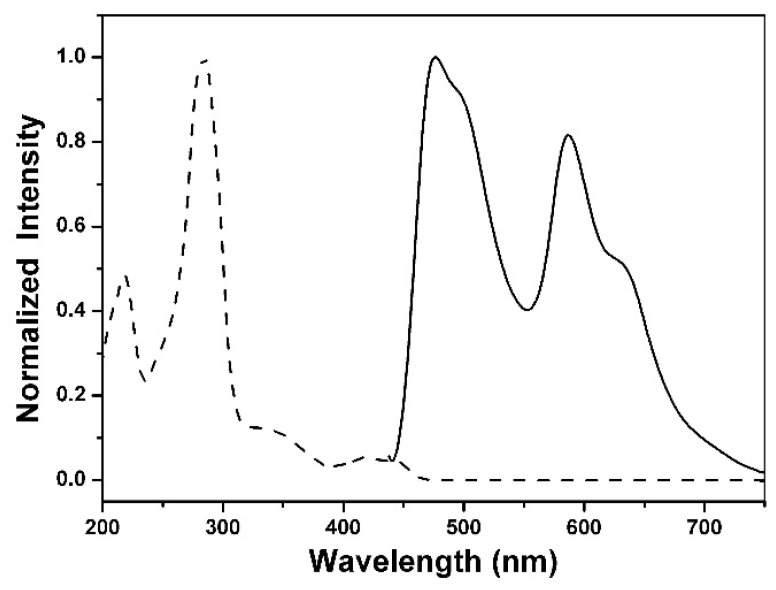
Normalized absorption (dashed line) and emission (solid line) spectra of **1** in ethyl acetate.

**Figure 6:**
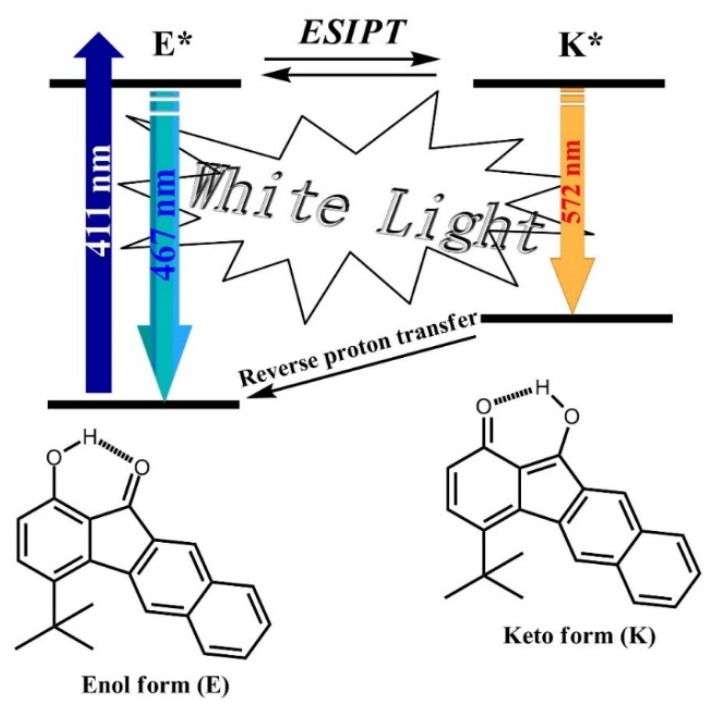
Schematic representation of the white-light generation process in **1**. ESIPT: excited-state intramolecular proton transfer.

Figure 5 shows the steady state absorption and emission spectra of **1** in ethyl acetate. Compound **1** exhibits the lowest lying absorption band maximized at 423 nm, attributed to a π → π* transition, which is also supported by the calculated frontier orbitals (*vide infra*). Additionally, the absorption spectrum of **1** is nearly identical with that of **2**, which demonstrates that the introduction of the *tert*-butyl group does not substantially affect the bandgap energy of **1** compared with that of **2**. As depicted in Figure 5, dual emission is well resolved in the steady-state measurement of **1**, which is composed of a normal emission band (enol form), justified by its mirror image with respect to the lowest lying absorption, and a large Stokes shifted (6605 cm^−1^) emission band maximized at 477 and 587 nm, respectively. Accordingly, the assignment of a 587 nm emission for **1** in ethyl acetate to a proton-transfer tautomer emission is unambiguous, and ESIPT takes place from the phenolic proton (O-H) to the carbonyl oxygen, forming the keto-tautomer species shown in Figure 6. Incidentally, the dual emission achieves a nearly white light generation with Commission Internationale de l’Eclairage (CIE) (0.35, 0.36). The overall quantum yield of **1** is measured to be 0.15 and is about four times larger than that of **2** (0.04), which can be explained by the fact that the bulky *tert*-butyl substituent reduces the intermolecular π-π stacking of **1** so that the quantum yield can be substantially enhanced.

### 3.5. Quantum Chemistry Computation

To gain more insight into the molecular structures and electronic properties of **1**, quantum mechanical calculations were performed using the density functional theory (DFT) at the B3LYP/6-31G** level. The values of bond lengths, bond angles, and torsion angles for **1** were compared with its crystal structure data. Table 2 compares the crystallographic and optimized geometric parameters of **1**. There are no substantial differences between the experimental and DFT/B3LYP calculated geometric parameters. Consequently, we can conclude that basis set 6-31G** is suited in its approach to the experimental results.

**Figure 7:**
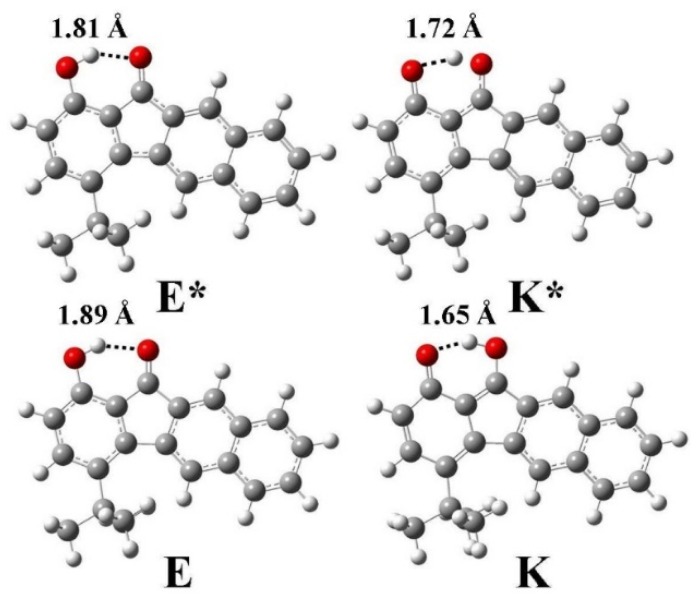
The optimized geometric structures of enol (E) and keto (K) form for **1** in the ground and the first singlet excited state together with the intramolecular hydrogen bond lengths.

The optimized geometric structures and the corresponding hydrogen bond lengths of enol and keto form for **1** in the ground and the first singlet excited state were calculated using DFT and TD-DFT with the B3LYP functional and the 6-31G** basis set (Figure 7). From E (K*) to E* (K), one can see that the intramolecular hydrogen bond length decreases from 1.89 (1.72) Å to 1.81 (1.65) Å, whereas the other bond lengths do not significantly change. The results clearly provide evidence for the strengthening of the intramolecular hydrogen bond from S_0_ → S_1_ (S_1_ → S_0_), which is consistent with previous studies [51,52,53]. Therefore, there is no question that the decreases of intramolecular hydrogen bond lengths from E (K*) to E* (K) is a very significant positive factor for the ESIPT (GSIPT: ground state intramolecular proton transfer) reaction.

**Figure 8:**
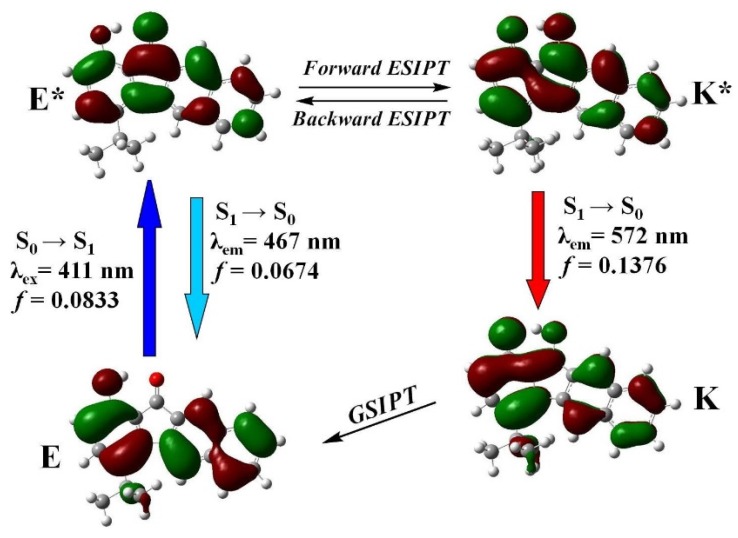
The frontier molecular orbitals of **1** for E, E*, K, and K*. GSIPT: ground state intramolecular proton transfer.

Figure 8 depicts the highest occupied molecular orbitals (HOMOs) and the lowest unoccupied molecular orbitals (LUMOs) of the enol and keto form of **1**, both of which are strongly delocalized over the entire π-conjugated system. It also shows that the electron density around the intramolecular hydrogen bonding system is mainly populated at hydroxyl oxygen and carbonyl oxygen at HOMO and LUMO, respectively. The results clearly show that upon electronic excitation of **1**, the hydroxyl proton (O(2)-H(2A)) is expected to be more acidic, whereas the carbonyl oxygen O(1) is more basic with respect to their ground state, driving the proton transfer reaction (forward ESIPT). After the forward ESIPT (E* → K*), the electron density located on O(2) increases while that on O(1) decreases, which shows the prominent intramolecular charge transfer from O(1) to O(2). This may supply the driving force for the proton transfer from O(1) to O(2) (backward ESIPT), so that the excited-state equilibrium can be established. In addition, the absorption and emission spectra of **1** were calculated by time-dependent DFT calculations (Franck-Condon principle, Figure 7). The calculated excitation, normal emission, and tautomer emission wavelengths for the S_0_ → S_1_ (S_1_ → S_0_) transitions are 411 nm, 467 nm, and 572 nm, respectively, which is very close to the experimental results (Figure 4).

**Figure 9:**
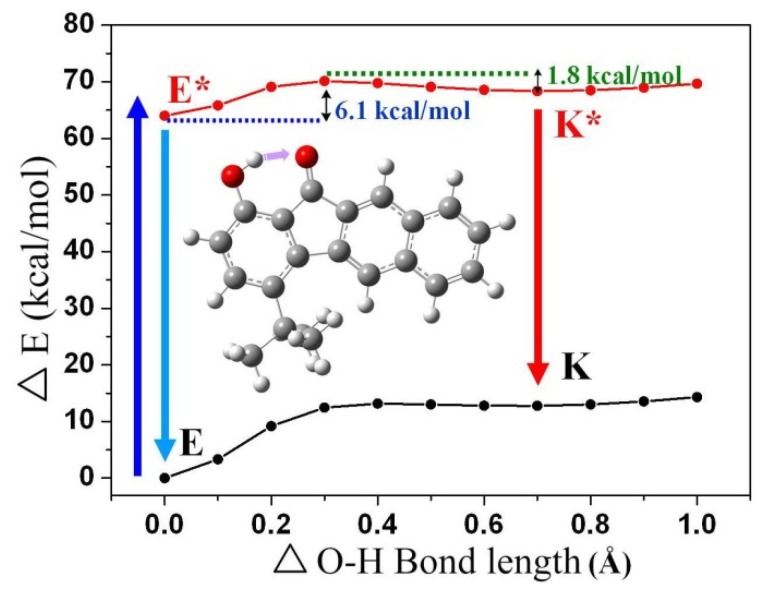
Potential energy curves (PECs) from enol form (E) to keto form (K) of **1** at the ground state and excited state. The calculations are based on the optimized ground state geometry (S_0_ state) at the B3LYP/6-31G**/ level using Gaussian 03W.

In order to explain the ESIPT properties of compound **1**, the potential energy curves of the intramolecular proton transfer as a function of the O(2)-H(2A) bond length (*i.e.*, the transformation from the enol form to the keto form) at both the ground state and the excited state were studied (Figure 9). On the one hand, the full geometry optimization based on the B3LYP/6-31G** theoretical level shows that the enol form (E) of **1** (**2**) in the ground state is more stable than the corresponding proton-transfer tautomer (K) by 12.8 (15.0) kcal/mol. As a result, proton transfer from K to E is populated in the ground states. It is also apparent that the increased phenolic (O(2)-H(2A)) acidity (hydrogen bonding strength, see 3.2) lowers the tautomerization energy by stabilizing the tautomers due to inductive effect of the bulky *tert*-butyl group. On the other hand (for the first singlet excited state), one can clearly see that the potential energy barriers of the forward (6.1 kcal/mol) and the backward (1.8 kcal/mol) ESIPT are in the same order of magnitude, which is in good agreement with previous theoretical studies of **2** [54]. Accordingly, the forward and the backward ESIPT may happen on a similar timescale, and hence leads to the rapidly established excited-state equilibrium.

### 3.6. Electrochemical Properties

**Figure 10:**
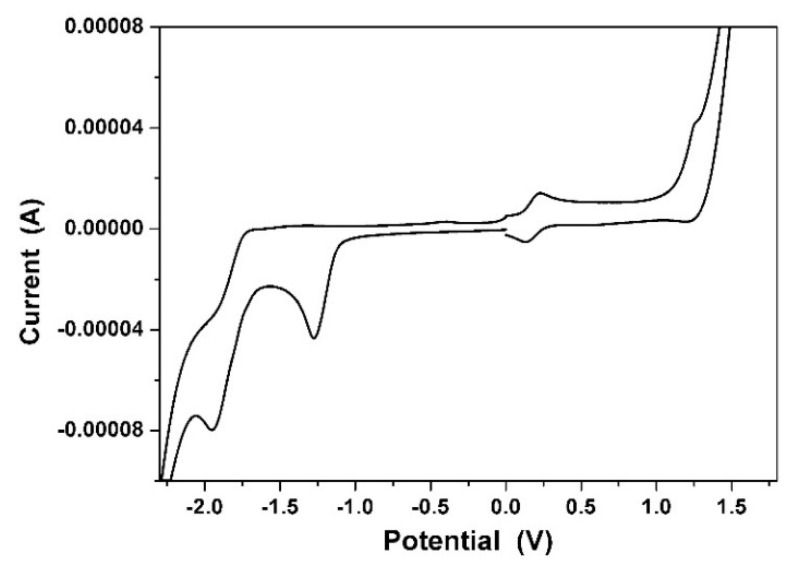
The cyclic voltammogram of **1** measured in dichloromethane solution with ferrocenium/ferrocene, at 200 mV/s.

Figure 10 shows the cyclic voltammogram of **1**. When placed in dichloromethane and subjected to modest potentials, compound **1** shows one oxidation and two reduction waves, all of which are chemically irreversible. The first oxidation and reduction potentials of **1** are almost identical to those of **2** (Table 3), showing that the alkylation of **2** has no significant impact on both their electrochemical properties as well as their optical properties. The redox potentials and the HOMO and LUMO energy levels estimated from cyclic voltammetry (CV) for **1** are summarized in Table 3. The HOMO/LUMO energy levels of **1** are estimated to be −5.87/−2.94 eV, and are in good agreement with the theoretical calculations.

**Table 3:** Calculated and experimental parameters for **1** and **2**.

Compound	λ_abs_^a^	λ_em_^a^	Φ ^b^	*E*_g_^c^	*E*(1) ^d^	*E*(-1) ^d^	*E*_HOMO_/*E*_LUMO_^e^	*E*_HOMO_/*E*_LUMO_^f^	*E*_g_^g^
**1**	423	477/587	0.15	2.93	1.30	−1.26	−5.87/−2.94	−5.91/−2.30	3.02
**2**	427	480/589	0.04	2.90	1.39	−1.28	−5.96/−3.06	−5.98/−2.31	3.00

^a^ Measured in ethyl acetate (in nm); ^b^ Determined with quinine sulphate as reference [45]; ^c^ At absorption maxima (*E*_g_ = 1240/λ_max_, in eV); ^d^ Measured in a solution of 0.1 M tetrabutylammonium hexafluorophosphate (TBAPF_6_) in dichloromethane *versus* SCE (in V); ^e^ Calculated from *E*_HOMO_ = −4.88 − (*E*_oxd_ − *E*_Fc/Fc+_), *E*_LUMO_ = *E*_HOMO_ + *E*_g_ (in eV); ^f^ Calculated by DFT/B3LYP (in eV); ^g^ Calculated by TD-DFT/B3LYP (in eV). HOMO: highest occupied molecular orbital; LUMO: lowest unoccupied molecular orbital.

## 4. Conclusions

In conclusion, we have successfully synthesized and characterized a new ESIPT-based white-light-emitting small molecule (**1**) with a bulky *tert*-butyl group. Compound **1**, as well as compound **2**, undergoes an intramolecular proton transfer reaction in the excited state, resulting in a tautomer that is in equilibrium with the normal species, exhibiting a dual emission that generates white light. The introduction of the *tert*-butyl substituent not only increases the solubility of **1** compared with **2**, but also improves the fluorescence intensity. Furthermore, analysis of the geometric structures clearly demonstrates that the intramolecular hydrogen bond length is shortened upon the photoexcitation, which is considered to be a very important factor for ESIPT. The potential energy curves demonstrate that the forward ESIPT and backward ESIPT may happen on a similar timescale and leads to the rapidly established excited-state equilibrium. Research on its application to single-molecule-based WOLEDs is currently in progress.
